# Vergiftungsanfragen aus Berlin und Brandenburg 1999–2018: Ein Stadt-Land-Vergleich

**DOI:** 10.1007/s00103-021-03305-0

**Published:** 2021-03-10

**Authors:** Franziska Thal, Thomas Reinhold

**Affiliations:** 1 Institut für Klinische Pharmakologie und Toxikologie – Giftnotruf, Charité – Universitätsmedizin Berlin, corporate member of Freie Universität Berlin, Humboldt-Universität zu Berlin, and Berlin Institute of Health, Berlin, Deutschland; 2 Institut für Sozialmedizin, Epidemiologie und Gesundheitsökonomie, Charité – Universitätsmedizin Berlin, corporate member of Freie Universität Berlin, Humboldt-Universität zu Berlin, and Berlin Institute of Health, Berlin, Deutschland

**Keywords:** Stadt-Land-Unterschiede, Intoxikation, Giftnotruf, Giftinformationszentrum, Vergiftung, Urban-rural differences, Intoxication, Poison control center, Poison information center, Poisoning

## Abstract

**Hintergrund und Ziel:**

Der Berliner Giftnotruf ist seit 1963 die zentrale Anlaufstelle beim Thema „Vergiftungen“ für die Berliner und Brandenburger Bevölkerung. Ferner nimmt die Einrichtung eine wichtige Funktion im Bereich der Vergiftungsprävention wahr. Ziel dieser Arbeit ist es, die Entwicklung des Beratungsaufkommens und der Inhalte von 1999 bis 2018 zu beschreiben. Unterschiede bei städtischer und ländlicher Herkunft der Anrufenden sowie bei privatem oder beruflichem Hintergrund der Anfragen werden betrachtet. Die Ergebnisse sollen der Verbesserung der Präventionsarbeit dienen.

**Methoden:**

Die Falldaten des Giftnotrufs (1999–2018) wurden aufbereitet und einer explorativen Datenanalyse unterzogen. Über Verfahren der deskriptiven Statistik wurden die Daten ausgewertet und analysiert. Zusammenhänge zwischen der „Herkunft des Anrufs“ (Stadt oder Land), dem „Hintergrund“ (privat oder beruflich) und der jeweiligen „Noxenkategorie“ wurden mittels Pearsons Chi-Quadrat-Test analysiert.

**Ergebnisse:**

Das jährliche Beratungsvolumen stieg tendenziell an. Insbesondere Anfragen zu Expositionen von Erwachsenen und Senioren nahmen zu. Häufigste Themen waren Vergiftungen mit Medikamenten und Publikumsmitteln. Anfragen zu illegalen Drogen nahmen am stärksten zu (durchschnittliche jährliche Wachstumsrate 6,3 %). Anfragenden Privatpersonen kann in den meisten Fällen direkt geholfen werden (86,8 %), sodass nur selten eine medizinische Behandlung empfohlen wird. Privatpersonen rufen häufiger aus der Stadt an, auf dem Land überwiegen Anrufe von medizinischem Personal. Im ländlichen Raum wurden Anfragen zu Schädlingsbekämpfungsmitteln, Pilzen, Tieren und Pflanzen häufiger gestellt. Anrufe zu Lebensmitteln, Fremdkörpern, Genussmitteln oder illegalen Drogen gingen hingegen vermehrt aus dem städtischen Raum ein.

## Abstract (en)

**Background and objective:**

Since 1963, the poison control center in Berlin has been the central helpline for the Berlin and Brandenburg population on the subject of poisoning. Furthermore, the institution performs a vital function in the field of poisoning prevention. The aim of this paper is to describe the development of the volume of consultations and their content from 1999 to 2018. Differences in the urban and rural origin of the callers as well as in the private or professional background of the inquiries are considered. The results will serve to improve prevention work.

**Methods:**

The case data of the poison control center (1999–2018) were evaluated and analyzed using descriptive statistical methods. Correlations between the categories “origin of call” (urban or rural area), “background” (private or professional), and “noxious agent” were analyzed using the Pearson’s chi-squared test.

**Results:**

The annual volume of consultations tended to increase. In particular, the increases are mainly related to inquiries regarding exposures of adults and seniors. The most frequent topics were poisoning with medications and products used in daily life. Inquiries about illegal drugs increased the most (average annual growth rate 6.3%). Inquiring persons with a private background can be helped directly in most cases (86.8%), so medical treatment is rarely recommended. Private persons call more frequently from urban areas, while calls from medical staff predominate in rural areas. Calls about pesticides, mushrooms, animals, and plants were more common in rural areas. Calls about food, foreign bodies, stimulants (alcoholic, caffeinated, and nicotine-containing foods/consumables), or illegal drugs, on the other hand, were received more frequently from urban areas.

## Hintergrund

Der Berliner Giftnotruf wurde 1963 in Anlehnung an die US-amerikanischen „Poison Control Centers“ als „Beratungsstelle für Vergiftungserscheinungen und Embryonaltoxikologie“ in der Städtischen Kinderklinik Charlottenburg gegründet. Im Jahr 1972 wurde er dem Bezirksamt Charlottenburg und 1992 der Berliner Senatsverwaltung für Gesundheit zugeordnet [1]. Der Zuständigkeitsbereich des Giftnotrufs erweiterte sich 1994 auf das Land Brandenburg, nachdem eine entsprechende Vereinbarung geschlossen wurde. Innerhalb der Berliner Senatsverwaltung wurde die Einrichtung 1995 in den Berliner Betrieb für Zentrale Gesundheitliche Aufgaben (BBGes) integriert. Nachdem die Embryonaltoxikologie im Jahr 2002 ein eigenständiger Fachbereich innerhalb des BBGes geworden war, führte man die Beratungsstelle für Vergiftungserscheinungen und den Fachbereich Klinische Toxikologie und Pharmakologie des BBGes im Jahr 2003 in einem Institut für Toxikologie zusammen [2, 3]. Um Synergieeffekte zwischen der Forschung, der klinischen Medizin und der über Jahrzehnte aufgebauten Erfahrung der Einrichtung nutzbar zu machen, wurde der Giftnotruf im Jahr 2012 in die Charité – Universitätsmedizin Berlin überführt. Seit Anfang 2019 ist der Giftnotruf innerhalb der Charité ein eigenständiger Arbeitsbereich des Instituts für Klinische Pharmakologie und Toxikologie [4], wodurch gezielt eine Schnittstelle zur Forschung und Lehre geschaffen wurde.

Der Giftnotruf in Berlin berät seit fast 60 Jahren Privatpersonen, Apotheken, Schulen, Kindergärten, die Polizei und Rettungskräfte sowie auch medizinisches Fachpersonal zu sämtlichen toxikologischen Fragestellungen. Mit jährlich ca. 45.000 Beratungen zu Humanexpositionen, ca. 1500 prophylaktischen Fragestellungen und ca. 500 Anrufen zu Vergiftungen bei (Haus‑)Tieren ist die Einrichtung bezogen auf die Beratungszahlen das am häufigsten frequentierte Giftinformationszentrum (GIZ) Deutschlands. Das offizielle Einzugsgebiet des Giftnotrufs der Charité ist mit den ca. 6 Mio. Einwohner*innen der Länder Berlin und Brandenburg das zweitkleinste der 8 deutschen Giftinformationszentren. Den Berliner Giftnotruf erreichen jedoch auch zahlreiche Anrufe von außerhalb des eigentlichen Einzugsgebiets und sogar aus dem Ausland.

Die rein telefonische Beratung erfolgt beim Giftnotruf der Charité im Rahmen eines 24-stündigen Schichtdienstes an 365 Tagen im Jahr. Auch wenn das Tagesgeschäft der Einrichtung in erster Linie durch Risikoeinschätzung und Beratung bei akuten Vergiftungs(verdachts)fällen geprägt ist, gehört auch die Aufklärung der Bevölkerung im Rahmen der Präventionsarbeit zu den Aufgaben des Giftnotrufs [5]. Die Information der Bevölkerung ist als besonders wichtiger Auftrag anzusehen, da hierdurch prospektiv auf das Vergiftungsgeschehen Einfluss genommen werden kann. Dabei ist es wichtig, die Intoxikationsvorfälle im jeweiligen Einzugsgebiet des Giftinformationszentrums über längere Zeiträume zu analysieren, um Trends und Neuerungen frühzeitig erkennen zu können und entsprechende Informationskampagnen abzuleiten. Diesem Erfordernis für die Bundesländer Berlin und Brandenburg nachzukommen, ist u. a. ein Ziel der vorliegenden Untersuchung.

Studien aus verschiedenen Ländern, wie z. B. den Vereinigten Staaten von Amerika [6–11], Polen [12, 13], Finnland [14], Weißrussland [15], China [16] und Afrika [17–19], haben gezeigt, dass sich Unterschiede bezüglich der Vergiftungsvorkommnisse im städtischen und ländlichen Raum feststellen lassen. Somit ist insbesondere die Variable „Herkunft des Anrufs“ (Stadt oder Land) im Rahmen einer zielgerichteten Aufklärungsarbeit zu berücksichtigen. Nach Kenntnis der Autorin und des Autors wurde für Deutschland zu diesem Thema bisher noch keine Studie veröffentlicht. Folglich stellt sich die Frage, ob sich auch hierzulande regionale Unterschiede zwischen Stadt und Land zeigen. Erkenntnisse darüber können beispielsweise bei der Umsetzung von zielgruppenorientierter Aufklärungsarbeit genutzt werden. Neben der longitudinalen deskriptiven Beschreibung der Anfragen aus den Bundesländern Berlin und Brandenburg steht demnach die Überprüfung zweier ausgewählter Arbeitshypothesen im Fokus der vorliegenden Arbeit:

### Hypothese 1.

Vergiftet sich eine Person auf dem Land, ist ein vergleichsweise längerer Anfahrtsweg zur nächstgelegenen Hausarztpraxis bzw. Rettungsstelle anzunehmen. Statistische Erhebungen zeigen, dass in vielen ländlichen Regionen die nächste Hausarztpraxis über 10 km und das nächste somatische (allgemeine) Krankenhaus bis zu 20 km entfernt sein können [20]. Aufgrund der geringeren medizinischen Anbindung auf dem Land [21] liegt die Vermutung nahe, dass Privatpersonen in ländlichen Regionen eher die telefonischen Angebote des Giftnotrufs nutzen als Privatpersonen im urbanen Raum.

### Hypothese 2.

Aus mehreren Studien geht hervor, dass sich Personen auf dem Land mit anderen Substanzen vergiften, als Stadtbewohner [6–8, 10–12, 14–19]. Dieser Zusammenhang wird auch in der vorliegenden Arbeit angenommen.

## Methoden

Grundlage der vorliegenden Untersuchung waren Falldaten des Giftnotrufs aus den Bundesländern Berlin und Brandenburg von 1999 bis 2018.

Für die Unterteilung der Falldaten in Anfragen aus ländlichen und urbanen Regionen wurde die Definition des Bundesinstitutes für Bau‑, Stadt- und Raumforschung (BBSR) herangezogen, wonach „… ein Stadt- oder Landkreis als ländlich [gilt], wenn … die Einwohner*innendichte im Gebiet unter 150 Einwohner*innen pro km^2^ [liegt]“ [22]. Dieser Definition zufolge gehört der Großteil Brandenburgs zum ländlichen Raum. Das Bundesland Berlin sowie alle Städte und Gemeinden im Bundesland Brandenburg mit ≥ 150 Einwohner*innen pro km^2^ [23] wurden zur „Stadt“ gezählt. Darüber hinaus wurden alle Gemeinden, welche offiziell zum Berliner Umland („Speckgürtel“) gehören [24], als urbanes Gebiet betrachtet und daher der „Stadt“ zugeordnet. Die einzelnen Städte und Gemeinden wurden anhand der im Giftnotruf dokumentierten Vorwahl identifiziert.

Jede Beratung im Giftnotruf der Charité wird über einen standardisierten Dokumentationsbogen protokolliert. Nach erfolgreicher Qualitätskontrolle hinsichtlich der Vollständigkeit und Plausibilität der dokumentierten Informationen werden die Daten digital erfasst und in einer Oracle-Datenbank gespeichert. Für die Auswertung der Falldaten war zunächst eine Datenharmonisierung erforderlich, da der standardisierte Protokollbogen über die letzten 2 Jahrzehnte geringfügige Modifikationen erfahren hat. Durch interne Interviews und eine systematische Analyse älterer und neuerer Falldaten konnten diese zeitweisen Anpassungen identifiziert und im Rahmen der Untersuchung berücksichtigt werden.

In einem nächsten Schritt wurden alle Fälle ohne genaue Ortsvorwahl, alle Anfragen präventiver Natur oder zu Vergiftungen bei (Haus‑)Tieren, Anrufe über vertraglich vereinbarte Rufumleitungen sowie alle Fälle, bei denen kein Gespräch zustande kam, ausgeschlossen. Im Ergebnis konnte in der Analyse eine Gesamtfallzahl von *n* = 251.554 berücksichtigt werden. Im Giftnotruf der Charité werden zusammengehörige Beratungen, welche denselben Vorfall betreffen, als „Fall“ zusammengeführt. Somit entspricht die genannte Stichprobengröße nicht der Gesamtzahl aller eingegangen Anrufe, sondern spiegelt die Anzahl an Fallkomplexen in der Datenbank wider.

Im darauffolgenden Arbeitsschritt wurde eine explorative Datenanalyse mit ersten deskriptiven Auswertungen über die „KNIME Analytics Platform“ (KNIME AG, Zürich, Schweiz) [25] durchgeführt. Für die bivariate Analyse der Zusammenhänge zwischen den Merkmalen „Herkunft des Anrufs“ (Stadt oder Land) und „Hintergrund der anfragenden Person“ (privat oder beruflich) bzw. „Herkunft des Anrufs“ und „Noxenkategorie“ wurde mittels der Software IBM SPSS Statistics V25 (IBM Corporation, Armonk, New York, USA) [26] ein Pearson χ^2^-Unabhängigkeitstest [27] durchgeführt und es wurden entsprechende Kreuztabellen erstellt. Da bei der Kategorie „Hintergrund der anfragenden Person“ (privat oder beruflich) insgesamt 101 Datensätze aufgrund fehlender Angaben zur anrufenden Person nicht verwertet werden konnten, betrug die Anzahl der hier ausgewerteten Fälle *n* = 251.453. Für die Auswertung des empfohlenen Prozederes nach „Hintergrund der anfragenden Person“ wurden die Anrufe von Privatpersonen unverändert übernommen und die ursprünglich erfassten Kategorien „Krankenhaus“, „Praxis“, „Rettungsdienst“, „anderes GIZ“ in der neuen Kategorie „Medizinisches Fachpersonal“ zusammengefasst. Unter der Kategorie „Weitere“ finden sich z. B. Anrufe der Polizei, anderer öffentlicher Institutionen oder von Pflegeheimen. Bei symptomlosen Expositionen bzw. leichten Vergiftungsfällen mit fehlender Dokumentation des angeratenen Prozederes wurden folgende Annahmen getroffen: Bei Anrufen aus Krankenhäusern oder Praxen entspricht das Prozedere bei fehlenden Werten „Geht nach Hause“. Werden Privatpersonen beraten, ist das weitere Vorgehen bei fehlenden Werten gleich „Bleibt zu Hause“.

Es werden 12 Noxenkategorien unterschieden: Medikamente, Publikumsmittel^1^, Pflanzen, Tiere, Pilze, Lebensmittel, Genussmittel, Chemikalien/berufliche Expositionen^2^, Fremdkörper, Drogen, Schädlingsbekämpfungsmittel und Sonstige^3^. Bei der Auswertung der Datenbankeinträge zur „Noxenkategorie“ galt es zu beachten, dass Mehrfachselektionen, z. B. bei Mischintoxikationen, möglich sind. Dementsprechend konnte es bei dieser Variablen vorkommen, dass bei der Auswertung eines Falls mehrere Noxenkategorien gleichzeitig angesprochen wurden. Im Rahmen der statistischen Testung wurden alle in der Datenbank erfassten Noxen berücksichtigt. Bei 566 Fällen war die Noxe nicht bekannt, sodass diese Fälle komplett ausgeschlossen werden mussten. Insgesamt konnten somit *n* = 250.988 Fälle mit 254.741 erfassten Noxen in die Auswertung einfließen. Die Stärke der Zusammenhänge zwischen den Variablen „Herkunft des Anrufs“ und „Hintergrund der anfragenden Person“ bzw. „Herkunft des Anrufs“ und „Noxenkategorie“ wurde über Cramers V ermittelt. Da es sich um eine explorative Datenanalyse handelte und keine Rückschlüsse von der Stichprobe auf die Grundgesamtheit gezogen werden sollten, wurde nicht für multiples Testen adjustiert.

## Ergebnisse

### Entwicklung der Vergiftungsanfragen 1999–2018

**Figure 1:**
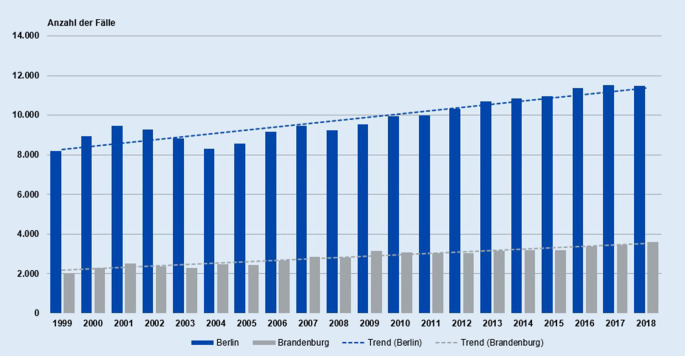

In den letzten 20 Jahren wurden beim Giftnotruf über 250.000 Fälle (Humanexpositionen) aus dem Einzugsgebiet Berlin-Brandenburg beraten. Den zeitlichen Verlauf betrachtend kann festgestellt werden, dass das jährliche Beratungsvolumen stetig zunahm. Während im Jahr 1999 noch 10.194 Beratungen durchgeführt wurden, waren es im Jahr 2018 bereits 15.084 (Abb. 1 und 2). Dies entspricht einer Zunahme um rund 48,0 %. Nur im Zeitraum von 2002–2004 und im Jahr 2008 sind Rückgange der Beratungszahlen festzustellen. Der Anteil der Ratsuchenden aus beiden Bundesländern blieb jedoch über alle Jahre hinweg mit ca. 76–80 % aus Berlin und ca. 20–24 % aus Brandenburg nahezu konstant.

**Figure 2:**
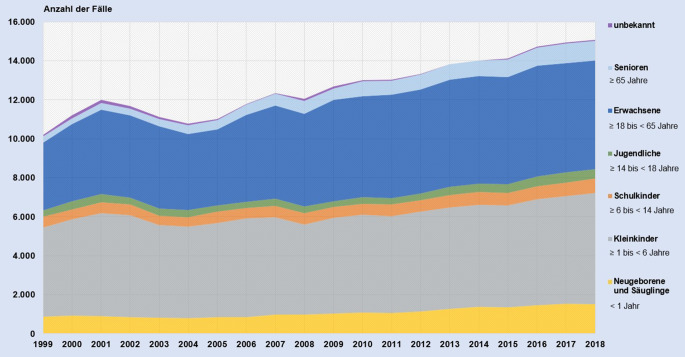

Bei den Altersgruppen der betroffenen Personen ist ein Trend erkennbar. Während sich die Anzahl der Anrufe zu Vergiftungs(verdachts)fällen bei Neugeborenen, Säuglingen, Kleinkindern, Schulkindern sowie Jugendlichen im hier untersuchten Einzugsgebiet des Giftnotrufs in den letzten 20 Jahren nicht erheblich gewandelt hat (+33,8 %), ist bei der Altersgruppe der Erwachsenen eine Steigerung der Beratungszahlen von 1999 bis 2018 um 60,3 % und bei den Senioren sogar um 220,1 % zu verzeichnen (Abb. 2).

**Figure 3:**
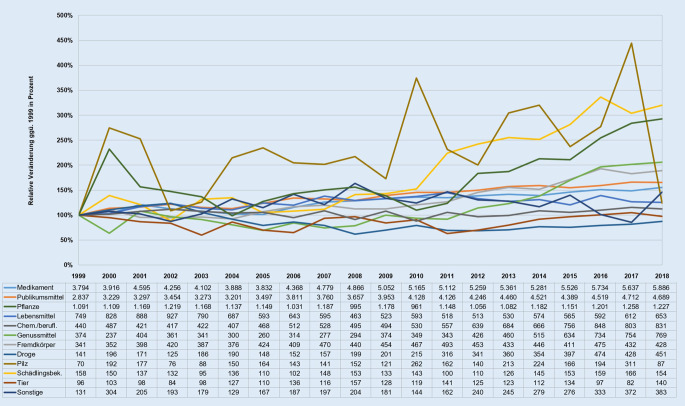

Im Giftnotruf der Charité werden alle Noxen kategorial in der Datenbank erfasst, sodass auch die Entwicklung der Anfragen zu den einzelnen Noxenkategorien über die vergangen 2 Jahrzehnte betrachtet werden kann. Deutlich zu erkennen ist hierbei, dass Medikamente und Publikumsmittel über alle Jahre hinweg am häufigsten in Vergiftungsfälle involviert waren (siehe Datentabelle in Abb. 3). In 7 von 10 Fällen fand im Giftnotruf eine Beratung zu Medikamenten oder Publikumsmitteln statt.

Bei fast allen Noxenkategorien ist über den Zeitverlauf eine positive durchschnittliche Wachstumsrate der Anfragen pro Jahr festzustellen (Abb. 3). Der größte jährliche Zuwachs ist dabei bei den illegalen Drogen (+6,31 %) zu verzeichnen. Lediglich bei den Kategorien „Lebensmittel“ und „Schädlingsbekämpfungsmittel“ ist mit negativen durchschnittlichen Wachstumsraten von −0,72 % bzw. −0,13 % pro Jahr ein leichter Rückgang zu beobachten. Die höchste Volatilität der Beratungszahlen liegt bei den Pilzen vor.

**Figure 4:**
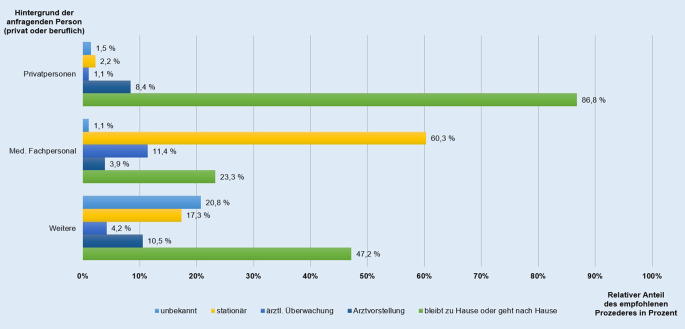

Zum Abschluss einer jeden Beratung im Giftnotruf wird eine Empfehlung für die weitere Vorgehensweise gegeben. Je nach Hintergrund der anfragenden Person (privat oder beruflich) differiert zumeist auch das empfohlene Prozedere (Abb. 4). Deutlich wird, dass Privatpersonen nach einer Beratung durch den Giftnotruf in den meisten Fällen keine weitere medizinische Behandlung empfohlen wurde. Der Mehrzahl von Privatpersonen aus Berlin und Brandenburg (86,8 %) konnte bezüglich der betroffenen Person geraten werden, zu Hause zu bleiben bzw. sich nach Hause zu begeben.

Auch wenn Personen in einer Arztpraxis vorstellig werden oder sich bereits in einer Klinik befinden, kann durch eine Beratung des Giftnotrufs relativ häufig (23,3 %) entschieden werden, dass die betroffenen Personen keiner weiteren ärztlichen Behandlung bedürfen (Abb. 4). Bei der Kategorie „Weitere“, innerhalb der alle Kontaktaufnahmen durch z. B. die Polizei, öffentliche Institutionen und Pflegeheime erfasst werden, liegt der Anteil der Betroffenen, welche zu Hause bleiben bzw. nach Hause gehen können, bei 47,2 %.

### Stadt-Land-Unterschiede

Die relativen Häufigkeiten der Beratungsfälle zeigen im Stadt-Land-Vergleich Besonderheiten. Bei der Variablen „Hintergrund der anfragenden Person“ ist auffällig, dass der relative Anteil anfragender Privatpersonen vom „Land“ 25,3 % niedriger liegt als bei den Anfragen aus der „Stadt“ (Tab. 1). Demgegenüber ist der relative Anteil des medizinischen Fachpersonals (Krankenhaus, Praxis, Rettungsdienst, anderes GIZ) vom Land deutlich erhöht. Insbesondere bei Rettungsdienstanfragen liegt der relative Anteil um 160,5 % höher im Vergleich zur Stadt. Mittels Pearsons χ^2^-Unabhängigkeitstest kann ein statistisch signifikanter Zusammenhang zwischen den Merkmalen „Herkunft des Anrufs“ (Stadt oder Land) und „Hintergrund der anfragenden Person“ (privat oder beruflich) gezeigt werden (*n* = 251.453; χ^2^ = 2072,846; df = 5; *p* < 0,0001; Cramers V = 0,091).

**Table 1:** 

		Hintergrund der anfragenden Person (privat oder beruflich)						
Herkunft des Anrufs^a^		Privat	Krankenhaus	Praxis	Rettungsdienst	Anderes GIZ	Weitere^b^	∑
Land	Anzahl	7895	7558	1236	609	76	501	17.875
	% innerhalb von Land	44,2	42,3	6,9	3,4	0,4	2,8	–
Stadt	Anzahl	138.060	76.345	8554	3055	573	6991	233.578
	% innerhalb von Stadt	59,1	32,7	3,7	1,3	0,2	3,0	–
–	∑ Anzahl	145.955	83.903	9790	3664	649	7492	251.453
	% Land gegenüber Stadt	−25,3	+29,4	+88,8	+160,5	+73,3	**−6,4**	–

*GIZ* Giftinformationszentrum

^a^ Bevölkerungszahlen für die beiden Kategorien („Stadt“ und „Land“) nach Stand der Gesamtbevölkerung von Berlin und Brandenburg 2017: „Land“: 1.087.973 Personen, „Stadt“: 5.029.562 Personen

^b^ Z. B. Polizei, öffentliche Institutionen und Pflegeheime

Bei der Betrachtung des Merkmals „Noxenkategorie“ und seiner relativen Häufigkeiten sind zwischen „Stadt“ und „Land“ (Tab. 2) ebenfalls Unterschiede ersichtlich. Hier zeigen sich für den ländlichen Raum gegenüber dem städtischen Gebiet höhere relative Anteile vor allem bei den Noxenkategorien „Schädlingsbekämpfungsmittel“ (+126,7 %), „Pilz“ (+100,1 %), „Tier“ (+49,2 %) sowie „Pflanze“ (+45,3 %). Einen geringeren relativen Anteil von Anfragen gegenüber der „Stadt“ kann hingegen bei den Noxenkategorien „Lebensmittel“ (−60,3 %), „Fremdkörper“ (−40,8 %), „Genussmittel“ (−33,1 %) und „Droge“ (−26,3 %) festgestellt werden. Der durchgeführte χ^2^-Unabhängigkeitstest sowie die Berechnung von Cramers V ergeben einen statistisch signifikanten, wenn auch schwachen Zusammenhang zwischen der Herkunft der anrufenden Person (Stadt oder Land) und der in den Vergiftungs(un)fall involvierten Noxenkategorie (*n* = 254.741; χ^2^ = 1413,817; df = 11; *p* < 0,0001; Cramers V = 0,074).

**Table 2:** 

			Noxenkategorie^b^											
Herkunft des Anrufs^a^		Publikumsmittel^c^	Medikament	Droge	Lebensmittel	Genussmittel	Fremdkörper	Chem./berufl.^d^	Schädlingsbekämpfungsmittel	Pflanze	Pilz	Tier	Sonstige^e^	∑
Land	Anzahl	4920	7336	276	377	413	367	872	397	2254	424	237	221	18.094
	% innerhalb von Land	27,2	40,5	1,5	2,1	2,3	2,0	4,8	2,2	12,5	2,3	1,3	1,2	–
Stadt	Anzahl	72.458	88.551	4896	12.412	8072	8106	10.467	2290	20.282	2772	2078	4263	236.647
	% innerhalb von Stadt	30,6	37,4	2,1	5,2	3,4	3,4	4,4	1,0	8,6	1,2	0,9	1,8	–
	∑ Anzahl	77.378	95.887	5172	12.789	8485	8473	11.339	2687	22.536	3196	2315	4484	254.741
	% Land gegenüber Stadt	−11,2	+8,4	−26,3	−60,3	−33,1	−40,8	+9,0	+126,7	+45,3	+100,1	+49,2	−32,2	–

^a^ Bevölkerungszahlen für die beiden Kategorien („Stadt“ und „Land“) nach Stand der Gesamtbevölkerung von Berlin und Brandenburg 2017: „Land“: 1.087.973 Personen, „Stadt“: 5.029.562 Personen

^b^ Mehrfachauswahl möglich: 250.988 ausgewertete Fälle mit 254.741 erfassten Noxen

^c^ Publikumsmittel: alle industriellen Erzeugnisse, mit denen Menschen außerhalb ihres Arbeitsumfeldes in Kontakt kommen (z. B. Wasch- und Reinigungsmittel), hier ohne Schädlingsbekämpfungsmittel, die separat erfasst werden

^d^ Chemikalien/berufliche Exposition

^e^ Sonstige: z. B. Kampfstoffe, Rauch, Dämpfe, kontaminiertes Wasser

## Diskussion

Ziel der Untersuchung war es, die Beratungsanfragen der letzten 20 Jahre in den Bundesländern Berlin und Brandenburg darzustellen, erste Anhaltspunkte bezüglich der möglichen Unterschiede beim Vergiftungsgeschehen in diesen Regionen zu erhalten und letztlich Implikationen für eine zielgruppenspezifischere Präventionsarbeit abzuleiten. Es konnte gezeigt werden, dass die Anfragen sowohl aus Berlin als auch aus Brandenburg über die letzten Jahre stetig zugenommen haben. Lediglich in den Jahren 2002–2004 und in 2008 waren Rückgänge zu beobachten. Als Ursache für den Rückgang 2002–2004 können strukturelle und personelle Veränderungen der Einrichtung in den Jahren 2002 und 2003 genannt werden [29], der Grund für die Abnahme im Jahr 2008 ist unbekannt.

Die insgesamt gestiegenen Beratungszahlen sind jedoch nicht notwendigerweise als eine generelle Zunahme von Vergiftungsvorkommnissen zu interpretieren, sondern können auch mit einem wachsenden Bekanntheitsgrad des Giftnotrufes erklärt werden. Auffällig ist hierbei jedoch, dass sich die Fallzahlen bei der Altersgruppe der „Erwachsenen“ und „Senioren“ in besonderem Maße erhöht haben. Ursächlich hierfür dürften u. a. die zunehmende Inanspruchnahme des Giftnotrufs durch Pflegeheime [30] sowie der demografische Wandel in Deutschland sein [31]. Aufgrund des demografischen Wandels rücken andere Einrichtungen, wie das Bundesinstitut für Risikobewertung (BfR), den Schutz älterer Menschen bereits mehr in den Mittelpunkt und weisen auf die spezifischen Risiken hin, denen Senioren ausgesetzt sind [32, 33]. Der Giftnotruf der Charité sollte dieser Entwicklung ebenfalls Rechnung tragen und seine Informationsmaterialien zielgruppengerecht anpassen.

Über den Zeitraum von 20 Jahren hinweg betrachtet wurde in 7 von 10 Fällen eine Beratung zu Vergiftungs(verdachts)fällen mit Medikamenten oder Publikumsmitteln durchgeführt. Diese Erkenntnis deckt sich auch mit den Beobachtungen anderer Giftinformationszentren [34, 35]. Demzufolge muss die Aufklärungsarbeit vor allem auf den korrekten Gebrauch und eine sichere Aufbewahrung von Medikamenten und Haushaltsprodukten ausgerichtet werden. Weiterhin konnte ein leichter Anstieg der Anfragen zu illegalen Drogen festgestellt werden, welche vermutlich mit dem generell steigenden Konsum von illegalen Rauschmitteln in Deutschland assoziiert ist [36]. Interessant ist in diesem Zusammenhang, dass Anfragen zu vermuteten Intoxikationen mit Drogen im analysierten Datensatz häufiger in städtischen Regionen dokumentiert sind. US-Studien haben jedoch gezeigt, dass tödliche Vergiftungsfälle mit Drogen schon längst nicht mehr nur ein Problem in Städten sind, sondern auch zunehmend in ländlichen Regionen auftreten [7, 10].

Die größten Schwankungen (Volatilität) der Beratungszahlen waren bei den Anfragen zu Pilzen zu beobachten. Diese können mit unterschiedlichen saisonalen Wetterverhältnissen erklärt werden, welche sich stark auf das Vorkommen einzelner Pilzsorten sowie auf den Ertrag und die Dauer der jeweiligen Pilzsaison auswirken [37]. Zur Vermeidung von Pilzvergiftungen wäre es für den Giftnotruf ratsam, sich zu Beginn der jährlichen Pilzsaison in Fachkreisen auszutauschen sowie das Wissen um die Gefahren des Verzehrs selbst gesammelter Pilze dauerhaft in die Bevölkerung zu tragen.

Beim empfohlenen Prozedere nach einer telefonischen Beratung zeigten sich Unterschiede im Hinblick auf den jeweiligen „Hintergrund der anfragenden Person“ (privat oder beruflich). So wurde bei 86,8 % der anrufenden Privatpersonen entschieden, dass keine weitere medizinische Behandlung der betroffenen Person notwendig ist. Stammte der Anruf von medizinischem Fachpersonal bzw. von „weiteren“ Personen waren es hingegen 23,3 % bzw. 47,2 %. Bei den Privatpersonen handelte es sich demnach in erster Linie um Expositionen, welche mit den richtigen Erstmaßnahmen leicht zu behandeln waren.

Da der Giftnotruf der Charité eine rein telefonische Beratungsstelle ist, kann bei einem erhöhten Anrufaufkommen wertvolle Zeit bis zur Empfehlung der Erstmaßnahmen verstreichen. Deshalb sollten Veröffentlichungen des Giftnotrufs für Privatpersonen Informationen zu den häufigsten Vergiftungs(verdachts)fällen sowie zu deren Behandlung enthalten (z. B. die häufig gestellten Fragen, FAQs). Darüber hinaus könnte das Onlineangebot der Einrichtung ausgebaut werden, um den Anforderungen der zunehmend digitalaffinen Nutzer*innen zukünftig gerechter zu werden.

Die Stadt-Land-Gegenüberstellung zeigte, dass sich die ursprüngliche Annahme, es würden mehr Privatpersonen aus dem ländlichen Raum den Giftnotruf nutzen (Hypothese 1), nicht bestätigt werden konnte. Obwohl die Stärke des Zusammenhangs mit einem Cramers V zwischen 0,05 und 0,1 schwach ausgeprägt gewesen ist [38], stellte sich heraus, dass tendenziell mehr Privatpersonen aus der Stadt anrufen. Im Gegensatz hierzu nehmen medizinische Fachkreise in ländlichen Regionen die Beratung des Giftnotrufs vergleichsweise häufiger in Anspruch, als ihre Kolleg*innen im städtischen Raum. Insbesondere der relativ höhere Anteil von Rettungsdienstanfragen aus der ländlichen Region war auffällig. Möglicherweise sind für den höheren Anteil des medizinischen Fachpersonals unter den Anfragenden die weiteren Anfahrtswege auf dem Land ursächlich, welche die Menschen dort häufiger veranlassen „sicherheitshalber“ den Rettungsdienst zu alarmieren oder direkt selbst die nächste Notaufnahme bzw. Arztpraxis aufzusuchen. Ein weiterer Erklärungsansatz für den verhältnismäßig geringeren Anteil der Privatanrufer*innen vom Land könnte auch der niedrigere Bekanntheitsgrad der Giftnotrufnummer bei der ländlichen Bevölkerung sein.

Im Rahmen des Stadt-Land-Vergleichs konnte weiterhin festgestellt werden, dass der Giftnotruf der Charité zu den Noxenkategorien „Schädlingsbekämpfungsmittel“, „Pilz“, „Tier“ sowie „Pflanze“ im Vergleich zur Stadt mehr Anfragen aus ländlichen Regionen erhält. Diese Unterschiede liegen vermutlich darin begründet, dass die Flora und Fauna zwischen Stadt und Land merklich differiert, Menschen in ländlichen Regionen sich womöglich mehr in der Natur aufhalten und im ländlichen Raum wesentlich mehr Pestizide eingesetzt werden [39]. Hingegen gehen aus dem urbanen Raum beispielsweise mehr Anrufe zu (verdorbenen) Lebensmitteln ein. So ist bekannt, dass die Stadtbevölkerung nachweislich einen schwächeren Bezug zur Urproduktion von Lebensmitteln hat und diese häufiger entsorgt als die Bewohner*innen ländlicher Regionen [40, 41]. Somit sind Stadtbewohner*innen vermutlich unsicherer, was den Umgang mit (verdorbenen) Lebensmitteln betrifft, wodurch sie den Giftnotruf diesbezüglich häufiger konsultieren.

Insgesamt konnten im Zuge des Stadt-Land-Vergleichs statistisch signifikante, wenn auch schwache Zusammenhänge zwischen den Merkmalen „Herkunft des Anrufs“ (Stadt oder Land) und „Hintergrund der anfragenden Person“ (privat oder beruflich) bzw. zwischen „Herkunft des Anrufs“ und der „Noxenkategorie“ nachgewiesen werden, was Hypothese 2 bestätigt.

Dieses Ergebnis belegt allerdings nur einen generellen Unterschied und erlaubt keine kausale Aussage darüber, welche der Kategorien der Merkmale „Hintergrund der anfragenden Person“ (privat oder beruflich) bzw. „Noxenkategorie“ konkret die Differenz zwischen Stadt und Land begründet. In internationalen Forschungsarbeiten wurde die Nutzung der Giftinformationszentren durch Personen mit unterschiedlichem Hintergrund (privat oder beruflich) in der Stadt und auf dem Land noch nicht untersucht. Bezüglich der Noxen konnten hingegen bereits einige Unterschiede belegt werden [6–8, 10, 11, 15, 19]. Hier ist jedoch noch mehr wissenschaftliche Aktivität erforderlich, um weitere, insbesondere neue Noxenkategorien abzudecken und die Übertragbarkeit der Ergebnisse auf den deutschsprachigen Raum überprüfen zu können.

Das Alter, das Geschlecht sowie die Vergiftungsumstände (Vergiftungsmodi) der betroffenen Personen unterscheiden sich anderen Forschungsarbeiten zufolge ebenfalls zwischen Stadt- und Landbevölkerung [12, 14, 16, 18]. Für Deutschland liegen hierzu bisher keine Erkenntnisse vor, weshalb auch an dieser Stelle zusätzlicher Forschungsbedarf besteht.

### Limitationen der Untersuchung

Eine Limitation des durchgeführten Stadt-Land-Vergleichs stellt die Tatsache dar, dass die ausgewerteten Fälle keiner repräsentativen Stichprobe entsprechen und lediglich die Tendenzen in der Region Berlin-Brandenburg widerspiegeln. Die Vergiftungs(verdachts)fälle anderer Großstädte und ländlicher Regionen in Deutschland konnten nicht in die Analyse einbezogen werden. Eine weitere Einschränkung liegt in dem Sachverhalt begründet, dass die Angabe zur „Herkunft des Anrufs“ im Giftnotruf der Charité lediglich zur anrufenden und nicht zur betroffenen Person erfasst wird. Dies kann insbesondere bei Anfragen durch medizinisches Fachpersonal, welches für seine Patient*innen anruft, zu Abweichungen führen. Da 99,1 % aller Berliner*innen und 63,5 % aller Brandenburger*innen ihr nächstgelegenes Krankenhaus jedoch innerhalb von 15 min Fahrzeit erreichen [42], dürften die hieraus folgenden Limitationen das Ergebnis der Analyse nicht relevant beeinflussen.

Darüber hinaus muss beachtet werden, dass nicht alle toxikologischen Beratungen der Berliner und Brandenburger Bevölkerung durch den Giftnotruf der Charité abgedeckt werden. Ein gewisser Teil wird auch durch andere Giftinformationszentren bedient. Um dieses Defizit auszugleichen, wäre es empfehlenswert, die Daten anderer Giftinformationszentren in zukünftige Studien einzubeziehen, um einen gepoolten Datensatz zu erhalten.

Ferner gehört Deutschland mit 232 Einwohner*innen je km^2^ im internationalen Vergleich zu den Staaten mit der größten Bevölkerungsdichte [43, 44]. Folglich dürften hierzulande die Stadt-Land-Unterschiede schwächer ausgeprägt sein, als in dünner und flächiger besiedelten Ländern, wie z. B. den USA mit 33 Einwohner*innen je km^2^ [45]. Dieser Umstand, aber auch unterschiedliche Stadt-Land-Definitionen sollten bei jeder Gegenüberstellung der Ergebnisse mit anderen Studien beachtet werden.

Eine weitere Limitation der Untersuchung stellt der sehr begrenzte Umfang der betrachteten Variablen dar. Um aussagekräftigere Ergebnisse zu erhalten, wäre eine umfassendere Analyse erforderlich, die ein breiteres Spektrum an zu untersuchenden Einflussfaktoren abdeckt. Sowohl teilweise lückenhafte Datenbankeinträge als auch das teils niedrige Skalenniveau der herangezogenen Daten sind weitere mögliche Schwächen der Analyse. Positiv festzuhalten ist allerdings die große Fallzahl des für die Analyse verwendeten Datensatzes sowie die vergleichsweise lange longitudinale Datenverfügbarkeit.

## Fazit

Die Vergiftungsanfragen aus dem Raum Berlin-Brandenburg wurden über einen Zeitraum von 20 Jahren betrachtet. Hierbei wurde erstmalig ein Stand-Land-Vergleich mit Daten eines in Deutschland ansässigen Giftnotrufs durchgeführt. Es konnten einige Tendenzen aufgezeigt werden, die Rückschlüsse auf das Vergiftungsgeschehen in den betrachteten Regionen zulassen. Weiterhin konnten Unterschiede zwischen den Anfragen aus der Stadt und denen aus dem ländlichen Raum dargestellt werden, sowohl im Hinblick auf den „Hintergrund der anfragenden Person“ (privat oder beruflich) als auch in Bezug auf die Noxenkategorie. Die nach erfolgter Beratung abgeleiteten Empfehlungen geben zudem Hinweise darauf, in welchem Ausmaß die Existenz eines Giftnotrufes möglicherweise unnötige Arztkontakte vermeidet und damit klassische medizinische Versorgungsstrukturen entlasten kann.

## Footnotes

1 Zu den Publikumsmitteln zählen definitionsgemäß alle industriellen Erzeugnisse, mit denen Menschen außerhalb ihres Arbeitsumfeldes in Kontakt kommen (z. B. Wasch- und Reinigungsmittel; [28]). Abweichend von dieser Definition werden im Giftnotruf der Charité Schädlingsbekämpfungsmittel nicht zu dieser Kategorie hinzugezählt und gesondert erfasst.

2 Der Kategorie „Chemikalien/berufliche Expositionen“ sind alle im beruflichen Kontext verwendeten Chemikalien/Substanzen (z. B. Industriereiniger) zugeordnet.

3 Die Kategorie „Sonstige“ fasst alle Noxen zusammen, die einer anderen Kategorie nicht sinnvoll zugeordnet werden konnten (z. B. Kampfstoffe, Rauch, Dämpfe, kontaminiertes Wasser).
